# Ovarian follicular dynamics, progesterone concentrations, pregnancy rates and transcriptional patterns in *Bos indicus* females with a high or low antral follicle count

**DOI:** 10.1038/s41598-020-76601-5

**Published:** 2020-11-11

**Authors:** Marina Amaro de Lima, Fábio Morotti, Bernardo Marcozzi Bayeux, Rômulo Germano de Rezende, Ramon Cesar Botigelli, Tiago Henrique Camara De Bem, Patrícia Kubo Fontes, Marcelo Fábio Gouveia Nogueira, Flávio Vieira Meirelles, Pietro Sampaio Baruselli, Juliano Coelho da Silveira, Felipe Perecin, Marcelo Marcondes Seneda

**Affiliations:** 1 Department of Veterinary Medicine, FZEA/USP, Pirassununga, SP Brazil; 2 Animal Reproduction and Biotechnology Laboratory, State University of Londrina-UEL, Londrina, PR Brazil; 3 Department of Animal Reproduction, FMVZ/USP, São Paulo, SP Brazil; 4 Institute of Biosciences, IBB/UNESP, Botucatu, SP Brazil; 5 Department of Biological Sciences, FCL/UNESP, Assis, SP Brazil; 6 Laboratório de Reprodução Animal, DCV, CCA, UEL, Cx. Postal: 10.011, Londrina, PR Cep: 86057-970 Brazil

**Keywords:** Cell biology, Molecular biology

## Abstract

We evaluated the effect of the antral follicle count (AFC) on ovarian follicular dynamics, pregnancy rates, progesterone concentrations, and transcriptional patterns of genes in Nelore cattle (*Bos taurus indicus*) after a timed artificial insemination (TAI) programme. Cows were separated based on the AFC, and those with a high AFC showed a larger (P < 0.0001) ovarian diameter and area than those with a very low AFC. Females with a very low AFC exhibited a larger (P < 0.01) diameter of the dominant follicle at TAI (13.6 ± 0.3 vs. 12.2 ± 0.4 mm) and a tendency (P = 0.06) to have different serum progesterone concentrations (2.9 ± 0.3 vs. 2.1 ± 0.3 ng/mL; on day 18, considering day 0 as the beginning of the synchronization protocol) than those with a high AFC. The pregnancy rate was higher (P ≤ 0.05) in animals with a very low (57.9%) and low (53.1%) AFC than in those with a high AFC (45.2%). The expression of genes related to intercellular communication, meiotic control, epigenetic modulation, cell division, follicular growth, cell maintenance, steroidogenesis and cellular stress response was assessed on day 5. In females with a low AFC, 8 and 21 genes in oocytes and cumulus cells, respectively, were upregulated (P < 0.05), while 3 and 6 genes in oocytes and cumulus cells, respectively, were downregulated. The results described here will help elucidate the differences in ovarian physiology and the reproductive success of *Bos indicus* females with a low or high AFC.

## Introduction

The relationship between the antral follicle count (AFC), reproductive performance and the efficiency of reproductive biotechniques in cattle has been the subject of numerous studies^1–11^. However, despite numerous advances, many aspects related to female reproductive physiology remain unknown, especially those related to differences between subspecies (*Bos taurus taurus* vs* Bos taurus indicus*), as well as the particularities related to the antral ovarian follicle population and its influence on cattle fertility^12–15^. The number of antral follicles is a highly variable characteristic in the bovine ovary^1–3,10^, but there is high repeatability in the follicular count in the same individual^1,10^. Due to high repeatability, taurine females were originally classified into low, intermediate, high, and very high AFC groups according to the number of antral follicles (follicles ≥ 3 mm). Such an assessment is performed by an ultrasound examination and does not vary independently because of the season, the number of follicular waves per oestrus cycle or the lactation status^1^. The same AFC repeatability was observed in *indicus-taurus* animals regardless of sexual maturity (weaning to yearling ages)^6,16^ and in *indicus* females during ovulation synchronization treatment for timed artificial insemination (TAI)^8^, a pharmacological strategy that allows the insemination of a large number of animals without the need for oestrus detection.

A low AFC in beef and dairy *taurus* females has been associated with several negative fertility aspects, such as small ovaries and a small number of morphologically healthy follicles and oocytes in ovarie^10^, poor reproductive performance at the end of the breeding season^17^, reduced responsiveness to superovulation treatment and a small proportion of transferable embryos^9,18^, low circulating progesterone (P4) and anti-Mullerian hormone concentrations^2,3,10^ and reduced endometrial thickness^19^. On the other hand, a high AFC has resulted in great efficiency in embryo production both in vivo and in vitro in *indicus* and *indicus-taurus* beef cattle^5,6,20^. However, studies on reproductive performance in Nelore cattle (*Bos indicus*) that received the TAI programme showed that the pregnancy rate for cows with a low AFC was up to 10% greater than that for cows with a high AFC^7,8^. In addition, Morotti et al.^8^ revealed that certain aspects of ovarian follicular dynamics, such as a large dominant and ovulatory follicle diameter when undergoing TAI programmes, are more favourable for the low AFC group. This positive correlation between dominant follicle size at the time of TAI and greater pregnancy rate is already well established^21–23^, although the specific reasons need to be better investigated at the cellular and molecular levels.

Surprisingly, in *taurus* dairy cattle, when heifers were monitored from sexual maturity to fifth lactation, it was revealed that a high AFC (≥ 25 follicles) resulted in a reduced productive life and suboptimal fertility compared to females with an AFC ≤ 15 follicles^4^. All these inconsistent results show how challenging this issue is, and many aspects and mechanisms of the relationship between the AFC and fertility remain unknown. A gene expression profile related to steroidogenesis, intercellular communication, meiotic control, epigenetic modulation, follicular growth, and cellular response to stress and apoptosis could better explain field fertility data. However, to date, most studies have been limited to practical field investigations, and there are no field fertility data showing the pattern of gene expression related to oocyte competence. In this context, the present study tested the hypothesis that a low AFC in Nelore females results in better fertility in the TAI programme than a high AFC. Therefore, the objectives of this study were as follows: (I) to evaluate the effect of a low/very low and high AFC on ovarian follicular dynamics and the pregnancy rate of cows submitted to the TAI programme and (II) to evaluate the transcriptional patterns of genes important to follicular cell development in heifers with low and high AFCs.

## Materials and methods

### Study I: Parameters of ovarian follicular dynamics and pregnancy rate in Nelore cows subjected to TAI

#### Ethics statement

This study was conducted according to the standards of the Ethics Committee for Animal Experimentation of the State University of Londrina under approval number 5898201476.

#### Location, animals and management

This study was performed during the usual time of beef cattle breeding (August to November) on two commercial beef farms in southern Brazil. The farms are located at a latitude of 23° 59′ 44″ S and a longitude of 51° 06′ 35″ W (Farm I) and a latitude of 24° 39′ 01″ S and a longitude of 50° 51′ 02″ W (Farm II). The climate in this region is Cfa according to Köppen-Geiger and characterized by a subtropical and humid clime, with an average temperature greater than 25 °C during the summer and a rainy season that extends from November to February, with over 1500 mm of rain precipitation.

Multiparous Nelore cows (*Bos indicus*; 48–84 months of age) were evaluated 40–50 days postpartum via a gynaecological examination and selected for two studies: ovarian follicular dynamics (*n* = 40; Farm I) and pregnancy rate to TAI (*n* = 1428; two herds from Farm II). The animals had a body condition score (BCS) between 2.5 and 4.0 on a scale of 1–5 (Lowman et al., 1976) and were maintained with continuous grazing of associated pastures of *Urochloa brizantha* and *Urochloa decumbens*. The management at both farms was similar, and all animals received mineralized mix and water ad libitum.

#### AFC and experimental design

The AFC was evaluated in each female prior to the ovulation synchronization protocol in the ovarian follicular dynamics study or on Day 0 for the TAI study. Then, the ovaries (right and left) of each female were scanned ultrasonically with a 7.5 MHz transducer (Aquila PRO, Pie Medical, Maastricht, the Netherlands), and antral follicles (all follicles ≥ 3 mm) were counted as previously described by Burns et al.^1^ and Ireland et al.^10^ to select the animals for the two experimental groups. Cows showing a very low AFC (VL-AFC, ≤ 15 follicles, *n* = 20) or high AFC (H-AFC, ≥ 45 follicles, *n* = 20) were intended to receive a conventional protocol of ovulation synchronization for TAI on the basis of P4 and oestrogen. All females with an AFC between 16 and 44 follicles were excluded from the ovarian follicular dynamics study.

**Figure 1:**
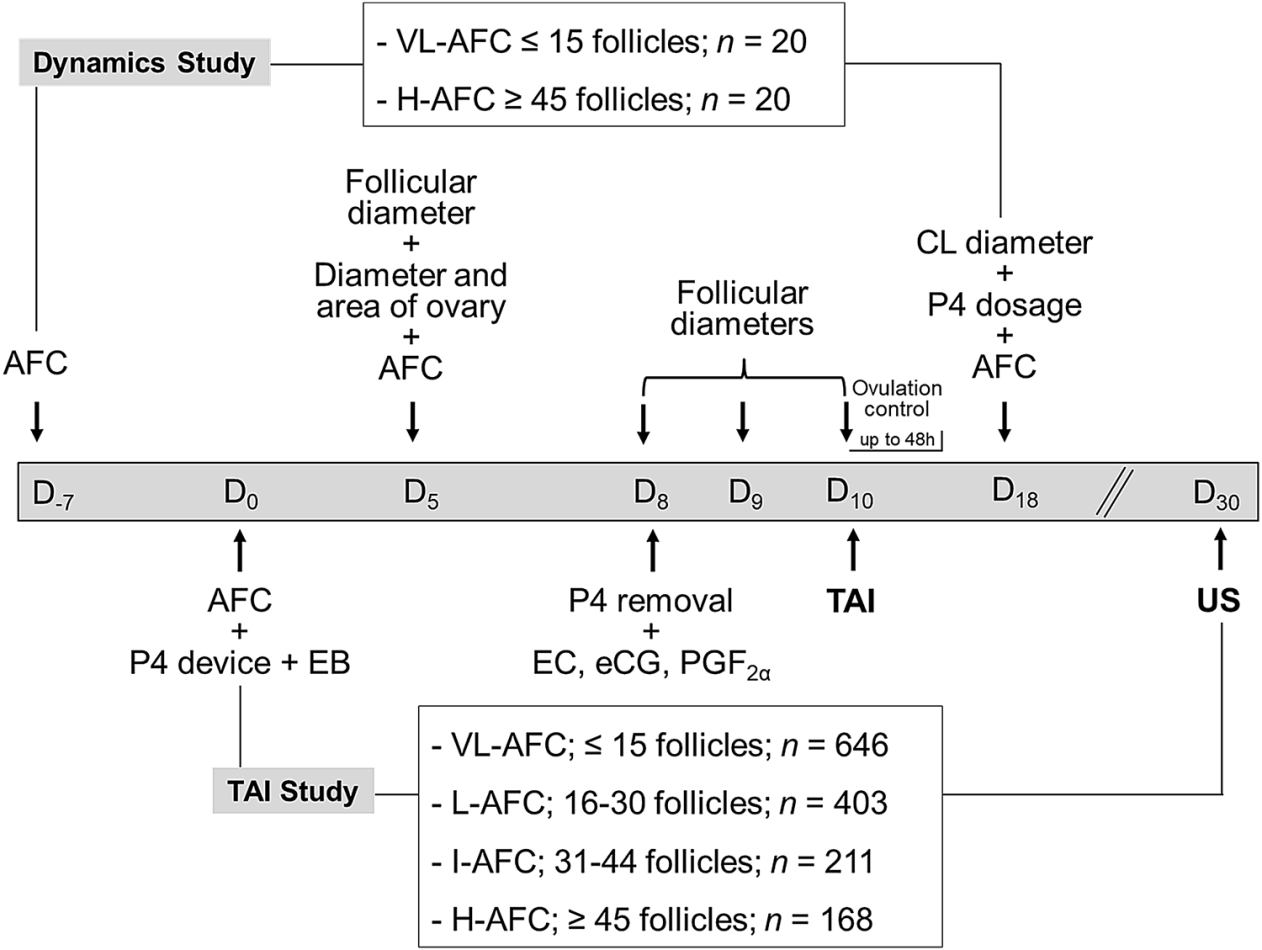
Experimental design used to evaluate ovarian follicular dynamics (*n* = 40) and pregnancy rate to timed artificial insemination (TAI; *n* = 1,428) in Nelore cows presenting different antral follicle counts (AFCs) and subjected to an ovulation synchronization protocol based on progesterone and oestrogen. *P4* progesterone, *EB* oestradiol benzoate, *EC* oestradiol cypionate, *eCG* equine chorionic gonadotrophin, *PGF2α* cloprostenol, *CL* corpus luteum, *US* ultrasound, *VL-AFC* very low AFC, *L-AFC* low AFC, *I-AFC* intermediate AFC, *H-AFC* high AFC.

To monitor ovarian follicular dynamics on a random day of the oestrus cycle (Day 0), 40 cows received an ear implant that contained 3 mg of norgestomet (Crestar, MSD Animal Health, Sao Paulo, Brazil) and an intramuscular (i.m.) administration of 2 mg of oestradiol benzoate (EB; Gonadiol, MSD). On day 8, the implants were removed, and the animals received i.m. administration that contained 250 μg of cloprostenol (PGF2α, Ciosin, MSD), 300 IU of equine chorionic gonadotropin (eCG; Novormon, MSD) and 1.0 mg of oestradiol cypionate (EC; ECP, Zoetis, Sao Paulo, Brazil), as illustrated in Fig. 1.

Performed by a single trained technician, ovarian follicular dynamics were assessed on days − 7, 0, 5, 8, 9, 10 and 18 (Fig. 1). The follicular diameters were determined on days 5, 8, 9 and 10; the AFC was determined on days − 7, 5 and 18; the area and diameter of the ovaries were determined on day 5; and the corpus luteum (CL) diameter was determined on day 18. The diameters were obtained from two transverse linear measurements of the follicular antrum (follicles) or the largest surface area of the evaluated structure (ovary or CL) after freezing of the image^24^.

The dominant follicle was defined as the follicle that on day 10 had a diameter ≥ 8 mm and exceeded the diameter of all other follicles^25^. The ovulatory follicle was considered the last diameter measured, with an interval of 12 h, before confirming ovulation. Ovulation was controlled by serial examinations every 12 h from day 10 and was verified by the absence of the dominant follicle previously recorded in the ovarian map and confirmed by the presence of the CL (7 days after the ovulation date) in the same ovary that contained the dominant follicle^24^. Immediately after CL measurement, jugular blood samples were collected and centrifuged for 15 min at 2.218 × *g*, and aliquots of serum were individually recovered in 3-mL polypropylene tubes and subsequently frozen at − 20 °C until the time of analysis. Serum P4 concentrations in 100-μL samples were determined using a commercial solid phase radioimmunoassay kit (RIA IM1188 kit; Beckman Coulter, Immunotech, Czech Republic). The sensitivity of the test was 0.1 ng/mL, and the intra-assay coefficients of variation were 3.0% for the highest value (24.240 ng/mL) and 0.1% for the lowest value (0.001 ng/mL).

To evaluate the pregnancy rate to TAI, a total of 1428 cows received the same hormonal application management, except the P4 source used was an intravaginal P4 device (DIB, Zoetis, Brazil), which remained from days 0 to 8 (Fig. 1). A single technician performed the insemination 48 h later with frozen-thawed semen from four bulls with fertility to TAI previously known.

### Study II: Transcriptional patterns of genes important to follicular cell development in Nelore heifers

#### Ethics statement

This study was approved by the Ethics Committee on Animal Use of the School of Veterinary Medicine and Animal Sciences, University of Sao Paulo, under protocol number 8968070518.

#### Location and animal management

The experiments were conducted at the University of Sao Paulo, Campus Fernando Costa, located in Pirassununga, Brazil, at a latitude of 21° 59′ 46″ S and a longitude of 47° 25′ 33″ W. Animals were handled in November 2016 during the usual season of beef cattle breeding in South America. At the location, this period coincides with the summer, with high rainfall levels and temperatures that usually vary between 20 and 30 °C. Heifers were maintained in a *Brachiaria brizantha* pasture, with protein-energetic supplementation (100 g/100 kg) and water available ad libitum.

#### Ovarian follicular wave synchronization and group formation

**Figure 2:**
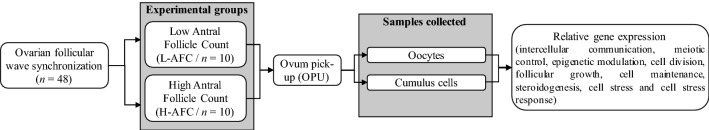
Experimental design and data collection. The ovarian follicular wave was synchronized in Nelore heifers (*n* = 48). On Day 5, an AFC (antral follicle count) was performed via an ultrasound examination. The females with the 10 lowest and 10 highest counts were assigned to the low AFC (L-AFC, number of follicles ≤ 29) and high AFC (H-AFC number of follicles ≥ 60) experimental groups, respectively. Additionally, on day 5, the follicular content with cumulus–oocyte complexes (COCs) was recovered via ovum pick-up (OPU). Cumulus cells and oocytes were separated from COCs by pipetting, and oocytes were denuded. Pools of 10 oocytes and pools of cumulus cells from 10 COCs from each animal were separately stored at − 80 °C until further analysis. Analyses included the determination of gene expression in oocytes and cumulus cells (genes related to intercellular communication, meiotic control, epigenetic modulation, cell division, follicular growth, cell maintenance, steroidogenesis, cell stress and cellular stress response).

On the first day (Day 0) of ovarian follicular synchronization, 48 Nelore heifers between 23 and 27 months of age and with body condition scores (BCS) between 3.5 and 4.5 (on a scale of 5) received 1 mg oestradiol benzoate (Sincrodiol, Ourofino Saude Animal) and an intravaginal progesterone device (Sincrogest, Ourofino Saude Animal). On Day 5, the device was removed, and an AFC was performed. To perform the AFC, transrectal ultrasonography with a linear array transducer (Mindray M5) was used to scan the ovaries (left and right) in each animal, and antral follicles (≥ 3 mm) were counted as previously described^1,10^. The 10 animals with the lowest (≤ 29 follicles) and 10 animals with the highest (≥ 60 follicles) AFCs were selected, resulting in two groups: heifers with a low AFC (L-AFC) and those with a high AFC (H-AFC; Fig. 2).

#### Ovum pick-up

On Day 5, cumulus-oocyte complexes (COCs) were recovered via ovum pick-up (OPU). Prior to OPU, the animals were subjected to epidural anaesthesia (3 mL of 2% lidocaine without a vasoconstrictor, 7 mg/kg; Lidovet, Bravet, Engenho Novo, RJ, Brazil), which was applied between the last sacral vertebra and the first coccygeal vertebra. After loss of tail reflexes, faeces were manually removed from the rectal ampulla, and the perineal and vulvar regions were sanitized with water. Then, a follicular aspiration guide (WTA, Cravinhos, SP, Brazil) with a micro convex 6.5 MHz probe (Mindray DP 2200) was introduced into the vaginal pouch. All the antral follicles in each animal were punctured with a disposable needle system (20 G, 0.9 × 40 mm; Terumo, Europe NV, Belgium) coupled to a Teflon aspiration line (1.7 mm). The aspiration system operated at a negative pressure between 12 and 15 mL of water/min (80–90 mmHg) produced by a vacuum pump. The material from the aspirated follicles was collected in a 50-mL conical tube (Corning) containing 15 mL of PBS (DPBS; Nutricell Nutrientes Celulares) and sodium heparin (5000 IU/L, Parinex, Hypolabor) that was maintained at 37 °C in a tube heater (WTA, Cravinhos, SP, Brazil) during OPU.

COCs and granulosa cells were collected from the tube content using a 75 µm IVF collector filter. COCs were selected, counted and separated in pools of 10 COCs of grades I, II and III with regard to the proportionality of their respective groups (L-AFC or H-AFC). Cumulus cells and oocytes were then separated by pipetting. Pools of 10 denuded oocytes and pools of cumulus cells from 10 COCs from each animal were separately stored at − 80 °C until further analysis.

#### RNA extraction and complementary DNA (cDNA) synthesis

Pools of 10 oocytes and pools of cumulus cells from 10 COCs were thawed and submitted to an RNA extraction protocol using TRI reagent (Molecular Research Center, Inc.) according to the manufacturer’s instructions with slight modifications. Total RNA was treated with DNAse I (Life Technologies) to eliminate eventual contamination with genomic DNA. cDNA was synthesized from total RNA of the pools using a High Capacity cDNA Reverse Transcription Kit (Applied Biosystems) according to the manufacturer’s instructions.

#### Gene expression quantification with a microfluidic system

The analysis of gene expression in bovine oocytes and cumulus cells from animals with low and high AFCs was performed using Applied Biosystems TaqMan Assays specific for *Bos taurus* in a microfluidic platform^26^. We analysed the mRNA abundance of 95 target genes (Supplementary Table 1) related to intercellular communication, meiotic control, epigenetic modulation, cell division, follicular growth, cell maintenance, steroidogenesis, cell stress and cellular stress response.

Prior to qPCR thermal cycling, each sample was submitted to a sequence-specific preamplification process as follows: 1.25 µL assay mix (Taqman Assay was pooled to a final concentration of 0.2× for each assay), 2.5 µL TaqMan PreAmp Master Mix (Applied Biosystems, #4391128) and 1.25 µL cDNA (5 ng/µL). The reactions were activated at 95 °C for 10 min, followed by denaturing at 95 °C for 15 s and annealing and amplification at 60 °C for 4 min for 14 cycles. These preamplified products were diluted fivefold prior to RT-qPCR analysis. For gene expression analysis, the sample solution prepared consisted of 2.25 µL cDNA (preamplified products), 2.5 µL TaqMan Universal PCR Master Mix (2×, Applied Biosystems) and 0.25 µL 20× GE Sample Loading Reagent (Fluidigm), and the assay solution consisted of 2.5 µL 20× TaqMan Gene Expression Assay (Applied Biosystems) and 2.5 µL 2× Assay Loading Reagent (Fluidigm). The 96.96 Dynamic Array Integrated Fluidic Circuits (Fluidigm) chip was used for data collection. After priming, the chip was loaded with 5 µL each assay solution and 5 µL each sample solution. qPCR thermal cycling was performed on a Biomark HD System (Fluidigm, South San Francisco, CA, USA) using the TaqMan GE 96 × 96 Standard protocol, which consisted of one stage of Thermal Mix (50 °C for 2 min, 70 °C for 20 min and 25 °C for 10 min) followed by a Hot Start stage (50 °C for 2 min and 95 °C for 10 min), followed by 40 cycles of denaturation (95 °C for 15 s) and primer annealing and extension (60 °C for 60 s). Three internal control genes (*RPL15, GAPDH* and *PPIA*) were evaluated using GeNorm software, and *PPIA* was selected as the internal control gene to normalize CT values. The expression values were calculated using the 2^−ΔCt^ method.

### Statistical analysis

In both studies, data from the very low or low AFC and high AFC groups were analysed for the normality of distribution using the Anderson–Darling test or the Shapiro–Wilk test and for homogeneity of variance using Levene’s test. Parametric variables were analysed with a two-tailed Student’s t-test for independent samples. Nonparametric variables were analysed with a Mann–Whitney test. The pregnancy rate was evaluated with the logistic regression model, including the AFC group as the main effect and the herd, BCS and bull as covariates. For descriptive analyses, the data are presented as the mean and standard error (M ± SEM) or percentage (%). For all statistical analyses performed, P ≤ 0.05 was considered to indicate significance unless otherwise stated.

## Results

### Study I: Ovarian follicular dynamics and pregnancy rate to TAI

The BCS, ovulation time and CL diameter after TAI were similar (P > 0.1) between cows with a very low or high AFC. Animals with a high count showed a greater (P < 0.0001) number of antral follicles and greater ovary diameter and ovarian area than those with a very low AFC on Day 5 of the TAI protocol. However, at the end of the hormonal protocol for TAI, cows with a very low AFC showed larger dominant follicle (P < 0.01) and tended to have larger preovulatory follicle (P = 0.06) diameters as well as higher serum P4 concentrations (P = 0.06; Table 1).

**Table 1:** Ovarian follicular dynamics data in Nelore cows with very low (VL-AFC; ≤ 15 follicles) and high (H-AFC; ≥ 45 follicles) antral follicle count (AFC; average of Days − 7, 5 and 18) evaluated during the protocol for timed artificial insemination (TAI).

Variables	VL-AFC (M ± SE)	H-AFC (M ± SE)	P-value
Animals (n)	20	20	–
AFC (n)	13.2 ± 0.9	50.3 ± 1.2	< 0.000
BCS (1–5)	2.9 ± 0.1	2.9 ± 0.1	0.74
Ovary diameter on Day 5 (mm)	21.1 ± 0.1	30.0 ± 0.1	< 0.0001
Ovary area on Day 5 (mm^2^)	39.2 ± 0.3	73.8 ± 0.3	< 0.0001
AFC by ovary area (follicles/mm^2^)	3.8 ± 0.4	7.0 ± 0.3	< 0.0001
Diameter of DF on Day 5 (mm)	7.9 ± 0.5	7.4 ± 0.3	0.41
Diameter of DF on Day 8 (mm)	11.5 ± 0.3	9.6 ± 0.4	0.001
Diameter of DF on Day 9 (mm)	12.5 ± 0.3	10.7 ± 0.4	0.001
Diameter of DF on Day 10 (mm)	13.6 ± 0.3	12.2 ± 0.4	0.008
Diameter of preovulatory follicle (mm)	14.6 ± 0.3	13.5 ± 0.5	0.06
Ovulation time (h)	70.2 ± 1.3	69.9 ± 1.6	0.81
CL diameter on Day 18 (mm)	19.1 ± 0.6	18.5 ± 0.3	0.53
P4 concentration on Day 18 (ng/mL)	2.9 ± 0.3	2.1 ± 0.3	0.06

*AFC* antral follicle count (values represent the average of Days − 7, 5 and 18), *VL-AFC* very low AFC, *H-AFC* high AFC, *BCS* body condition score, *DF *dominant follicle, *CL* corpus luteum, *P4* progesterone.

The number of antral follicles varied (P < 0.0001) according to the AFC group (VL-AFC, L-AFC, I-AFC and H-AFC), and the lowest TAI pregnancy rate (P = 0.001) was found in cows with a high count (H-AFC) compared with groups with fewer antral follicles (VL-AFC, L-AFC and I-AFC; Table 2). The pregnancy rate to TAI was not influenced by herd (P = 0.73), BCS (P = 0.48) or bull (P = 0.18).

**Table 2:** Average antral follicle count (AFC) and pregnancy rate from multiparous Nelore cows with a very low (VL-AFC; ≤ 15 follicles), low (L-AFC; 16–30 follicles), intermediate (I-AFC; 31–44 follicles) and high (H-AFC; ≥ 45 follicles) AFC subjected to a timed artificial insemination (TAI) programme.

AFC group	Animals	AFC (M ± SE)	Pregnancy rate % (n)
Very low (≤ 15 follicles)	646	10.6 ± 0.2^d^	57.9ª (374)
Low (16–30 follicles)	403	23.9 ± 0.2^c^	53.1^a^ (214)
Intermediate (31–44 follicles)	211	37.3 ± 0.3^b^	54.9ª (116)
High (≥ 45 follicles)	168	53.1 ± 0.6^a^	45.2^b^ (76)
Total/P-value	1428	< 0.0001	0.001

*AFC* antral follicle count represents the average of groups determined on Day 0 of the TAI protocol.

Different lowercase letters (a–d) in the same column indicate differences.

### Study II: Transcriptional patterns of genes important for follicular cell development in Nelore heifers

#### AFC and recovery of cumulus–oocyte complexes

**Figure 3:**
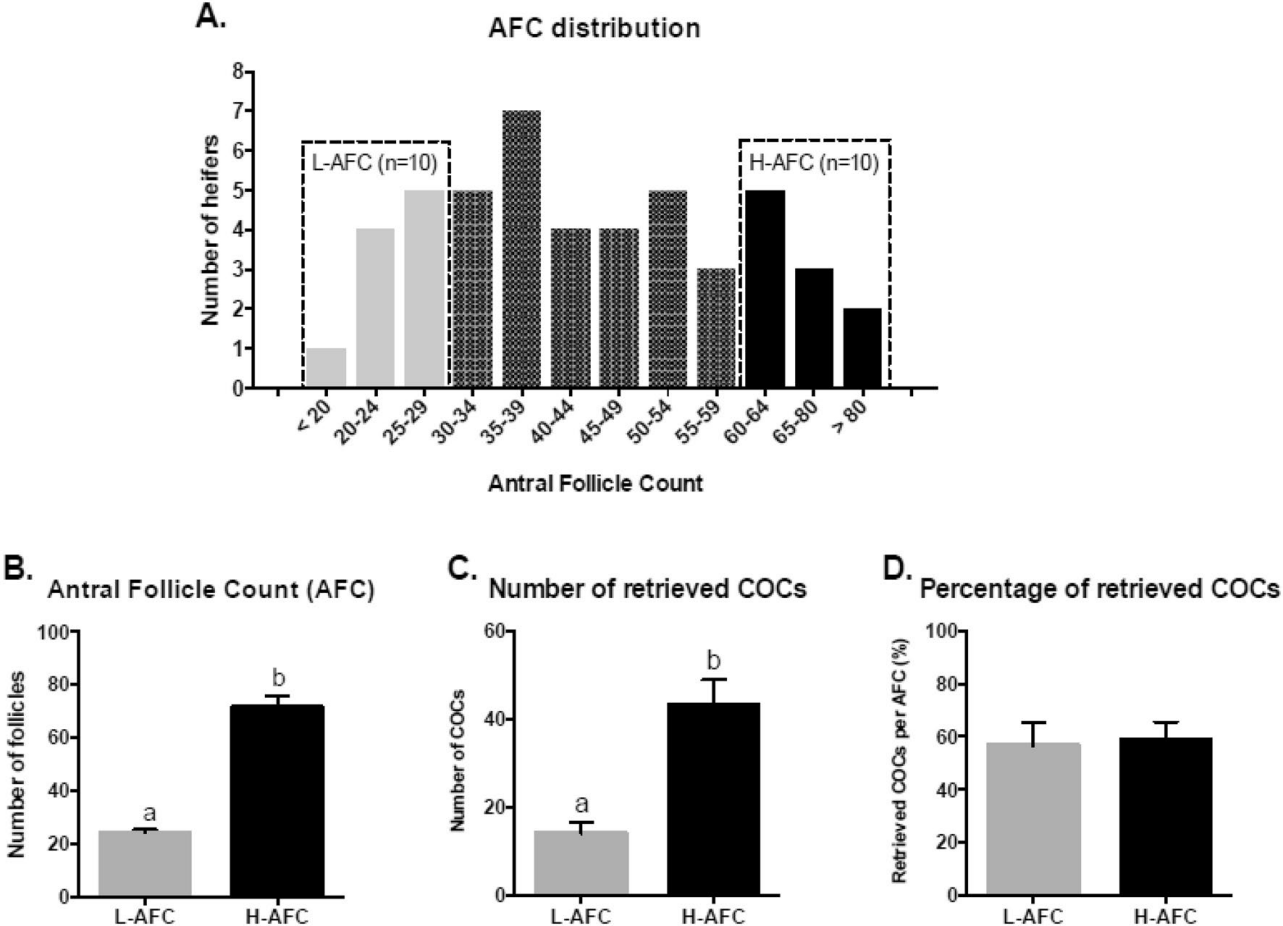
Antral follicle count (AFC) and COC recovery on day 5 of ovarian follicular wave synchronization protocol (day 0 = P4 device insertion) in the L-AFC and H-AFC groups. (**A**) Histogram presenting the frequency distribution of the antral follicle count in the 48 heifers evaluated. Dashed rectangles indicate the bottom 10 (L-AFC) and top 10 (H-AFC) animals, which formed the experimental groups. (**B**) Number of follicles; (**C**) number of retrieved COCs; (**D**) and percentage (number of COCs/number of follicles × 100) of COCs retrieved via OPU in heifers with a low (*n* = 10) or high (*n* = 10) AFC. Different letters (a and b) indicate significant differences (P ≤ 0.05). Grey and black bars represent the means of the L-AFC and H-AFC groups, respectively. Bars represent the mean, and error bars represent the standard error of the mean.

After determining the AFC in 48 Nelore heifers, the experimental group of low AFC animals was formed from the 10 animals with the lowest number of follicles (L-AFC, follicle number ≤ 29, AFC = 24.0 ± 4.7, range 14–29 follicles), and the experimental group of high AFC animals was formed from the 10 animals with the highest number of follicles (H-AFC, number of follicles ≥ 60, AFC = 72.3 ± 15.7, range 60–107 follicles) (Fig. 3A,B). The number and percentage of COCs recovered via OPU from the animals in the two groups were also determined. L-AFC animals presented a smaller (P ≤ 0.05) number of retrieved COCs than H-AFC animals (14.1 COCs ± 8.2 and 43.2 COCs ± 18.3, respectively; Fig. 3C), but the percentage of retrieved COCs was similar (P > 0.05) among the L-AFC and H-AFC animals (58.8% ± 21.3 and 56.6% ± 26.8, respectively; Fig. 3D).

#### The AFC is associated with transcriptional patterns in different compartments of the ovarian follicular microenvironment

**Figure 4:**
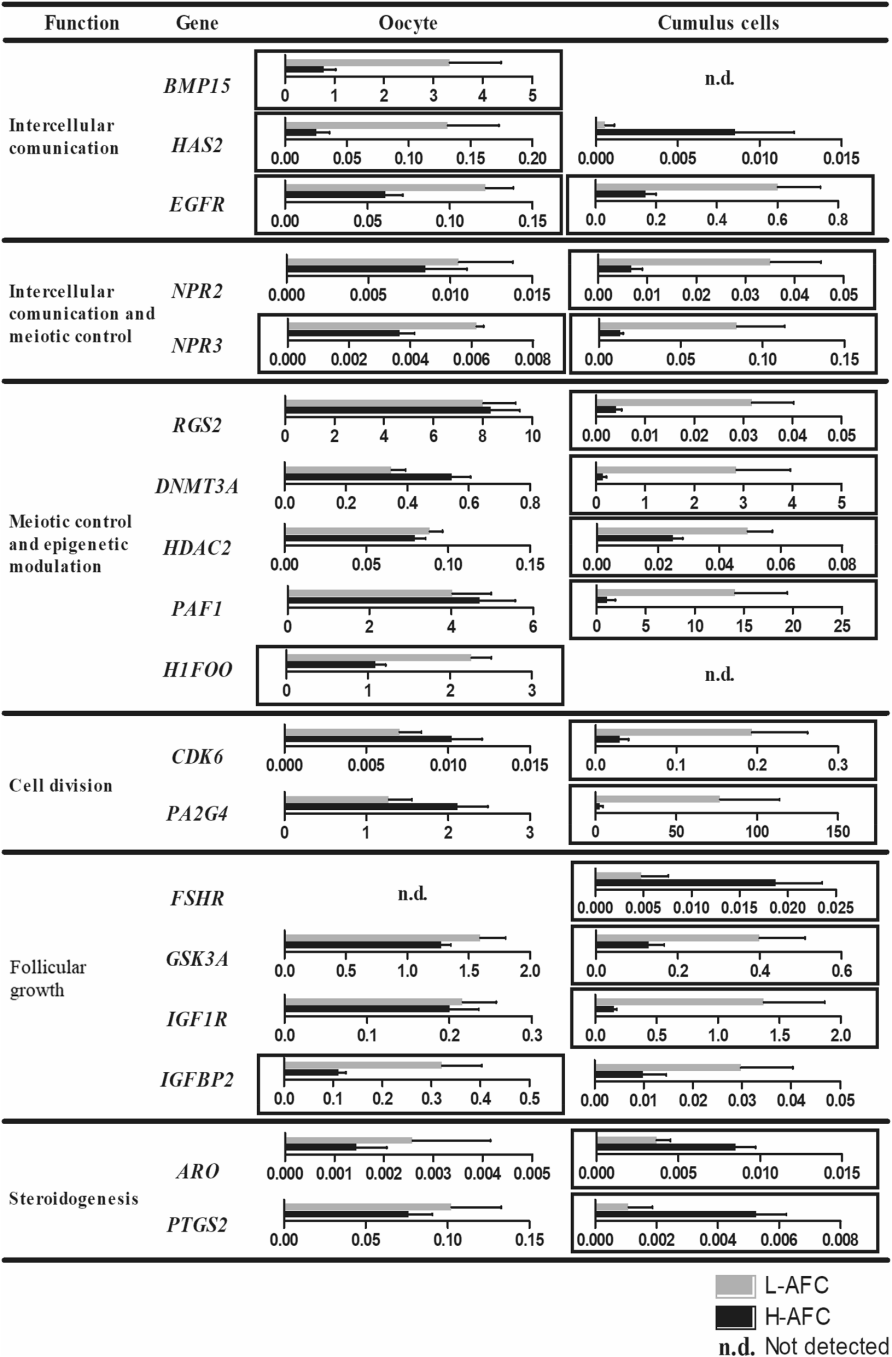

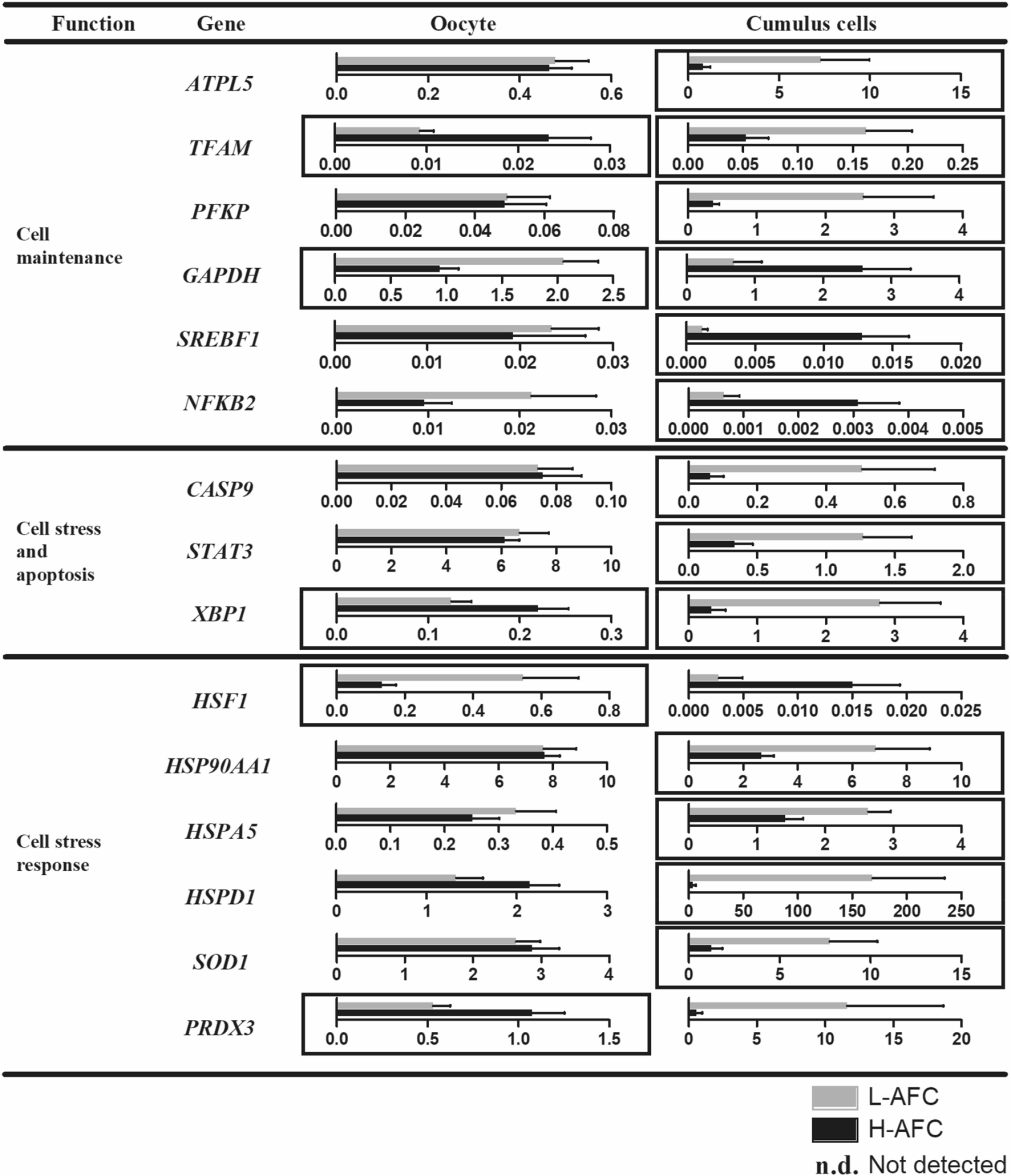
Relative expression of differentially expressed genes in oocytes and cumulus cells from L-AFC or H-AFC Nelore heifers. COCs were collected for follicular aspiration 5 days after intravaginal P4 device insertion and estradiol benzoate treatment*.* The genes are grouped by function as follows: intercellular communication, meiotic control, epigenetic modulation, cell division, follicular growth, cell maintenance, steroidogenesis, cell stress and cell stress response. The L-AFC group is represented by grey bars; the H-AFC group is represented by black bars. Significant differences (P ≤ 0.05) are indicated by a black rectangle surrounding each graph. Bars represent the mean, and error bars represent the standard error of the mean.

To evaluate whether the transcriptional patterns of genes important for follicular cell development differ between L-AFC and H-AFC oocytes and cumulus cells, 95 genes with different cellular functions were evaluated. Among the studied transcripts were genes involved in intercellular communication, meiotic control, epigenetic modulation, cell division, follicular growth, cell maintenance, steroidogenesis, cell stress and cellular stress response. The relative expression of the differentially expressed genes in oocytes and cumulus cells from L-AFC and H-AFC heifers is shown in Fig. 4. The mean, standard deviation, number of samples with expression detected and P-values for all the genes analysed in oocytes and cumulus cells from the L-AFC and H-AFC groups are shown in Supplementary Table 2.

In oocytes, a total of 11 genes were differentially expressed between the groups. Genes involved in intercellular communication (*BMP15, HAS2* and *EGFR*), intercellular communication involved in meiotic control (*NPR3*), epigenetic modulation (*H1FOO*) and follicular growth (*IGFBP2*) were upregulated in the L-AFC group. In the same group, some genes related to cell maintenance and response to cell stress (*GAPDH* and *TFAM*, respectively) were upregulated, whereas others (*HSF1* and *PRDX3*, respectively) were downregulated, while the *XBP1* gene, which is related to cell stress and apoptosis, showed reduced expression compared with that in the H-AFC group.

Cumulus cells were the intrafollicular compartment most affected by AFC, presenting 27 genes differentially expressed between the groups. As in oocytes, cumulus cells from L-AFC animals showed upregulation of genes associated with intercellular communication (*EGFR*), intercellular communication involved in meiotic control (*NPR3* and *NPR2*) and epigenetic modulation (*DNMT3A*, *HDAC2*, *PAF1*) but also showed increased expression of genes related to cell division (*CDK6*, *PA2G4*), cell stress and apoptosis (*CASP9*, *STAT3*, *XBP1*) and stress response (*HSP90AA1*, *HSPA5*, *HSPD1*, *SOD1*). Up- and downregulation of gene expression related to follicular growth functions (*IGF1R* and *GSK3A* genes were increased, while the *FSHR* gene was decreased) and cell maintenance (*ATPL5*, *TFAM* and *PFKP* genes were upregulated while *GAPDH*, *SREBF1* and *NFKB2* were downregulated) was observed in the L-AFC cumulus cell group. Genes related to steroidogenesis (*ARO*, *PTGS2*) were found to have lower expression patterns in L-AFC animals than in H-AFC animals.

## Discussion

This study provides the first description of the relationship between in vivo fertility and the AFC in *Bos indicus* cattle, supported by the results of a field investigation and a gene expression panel containing genes considered important to follicular cell development, oocyte competence and female fertility. Initially, it was found that females with a low AFC presented large follicular diameters when submitted to TAI, resulting not only in a large diameter of the dominant and ovulatory follicle but also in a tendency to have higher P4 concentration. At the same time, it was evidenced that the pregnancy rate in females with a low AFC is more than 10% that of those with a very high AFC when submitted to the TAI programme.

**Figure 5:**
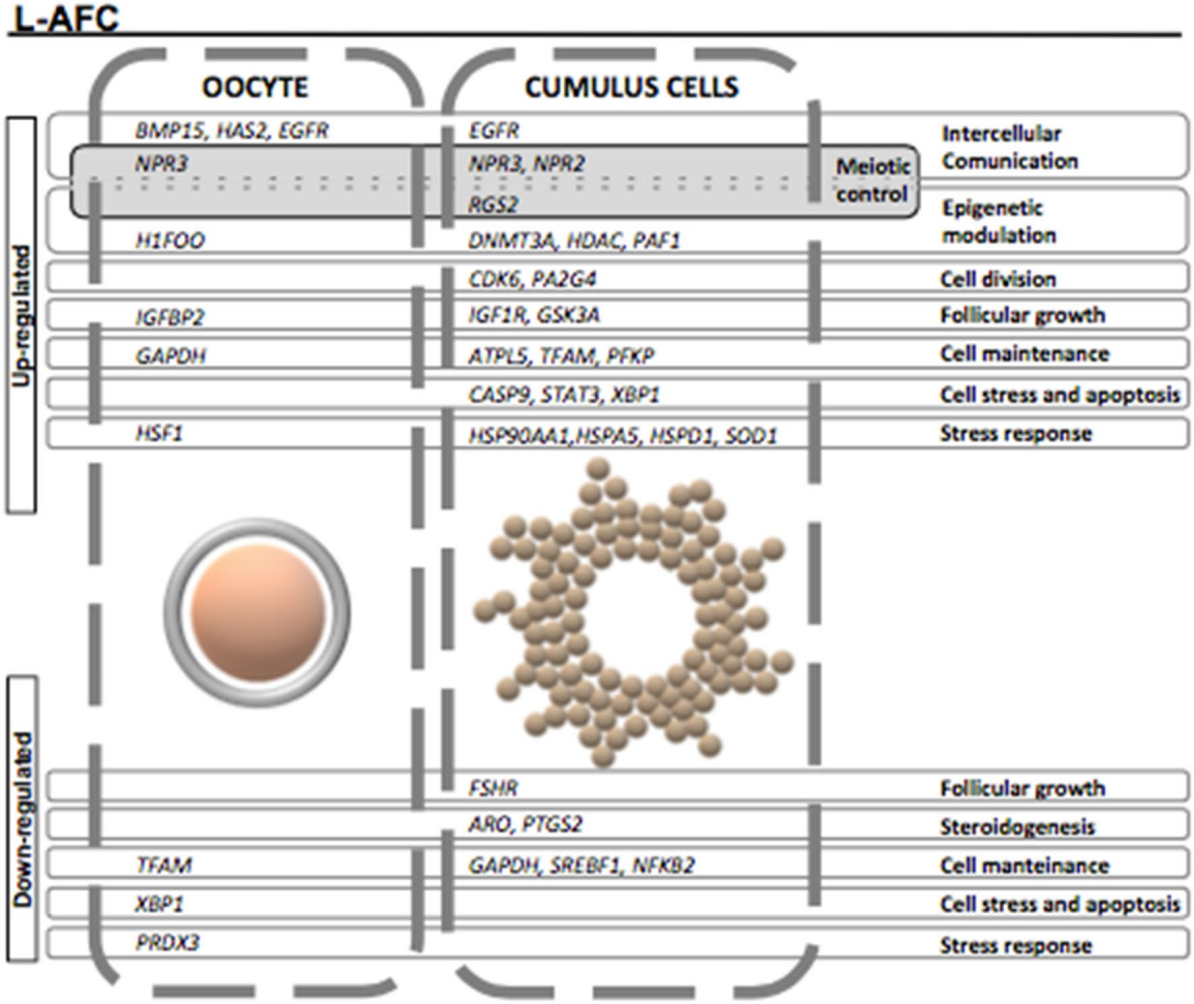
Genes differentially expressed in oocytes and cumulus cells from heifers with a low antral follicle count (L-AFC) or high AFC (H-AFC). Differences are indicated as genes upregulated or downregulated in the L-AFC group compared with the H-AFC group. Up- or downregulation is indicated on the left. Cell types (oocyte or cumulus cells) are isolated by dashed rectangles. Differentially expressed genes are in the dashed rectangle of the respective cell type. Solid-line rectangles correspond to gene functions, as shown to the right.

The present study also showed that important genes linked to oocyte, cumulus cell and follicular fluid functions (intercellular communication, meiosis control, epigenetic modulation, cell division, follicular growth, steroidogenesis, cell maintenance and stress—summarized in Fig. 5) are differently expressed in animals with a low compared with a high AFC. Finally, this study reports a relevant description that supports, on a molecular basis, greater TAI fertility in Nelore (*Bos indicus*) cows with a low AFC, corroborating recent publications on the relationship between fertility and AFC in cattle^4,7,8^.

The ovarian size (diameter and ovarian area) in this study showed that cows with a high AFC present larger measurement than cows with a low AFC. Similar data were reported by Morotti et al.^8^ in *Bos indicus* cattle, showing greater diameter (high 28.3 ± 3.9 vs. low 20.5 ± 3.2 mm) and greater ovarian area (high 67.5 ± 16.4 vs. low 37.7 ± 11.7 mm^2^) in cows with a high AFC than in those with a low AFC. In *Bos taurus* cattle, Martinez et al.^27^ observed the same relationship between AFC and ovarian area (high 51.9 ± 12.9 vs. low 42.1 ± 15.2 mm^2^). Although this relationship may be well established in cattle, to confirm that the groups were well distributed in the present study, a relationship was established between the number of antral follicles of each female and the total area of the ovary. Such relationship showed that high AFC females had almost twice the number of follicles per ovarian area compared with the very low group (7.00 vs. 3.78 follicles/mm^2^, respectively; Table 1).

A strategic aspect of this study was the evaluation of the diameter of the dominant follicle that on the day of emergence was similar between the groups. However, the diameter of the dominant follicle was greater for cows with a very low AFC than for those with a high AFC from the day of removal of the P4 device (Day 8 of the TAI protocol) until ovulation time. Similar data were published by Morotti, et al.^8^ who associated a low AFC with the larger follicular diameters throughout the TAI protocol and greater pregnancy rate in relation to high AFC (61.7 vs. 49.5%). In this context, the size of the dominant follicle at the time of TAI is widely related to many positive aspects of female fertility^21,28,29^. It has been reported that the largest diameter of the dominant follicle at TAI is associated with greater oestrus expression^21,30^, ovulatory rate^21,31^, CL size and P4 concentration^22^, and pregnancy probability^23,28,32^.

In this context, the field data from this study on both ovarian follicular dynamics and TAI fertility confirm the hypothesis that low-AFC Nelore cows show higher reproductive performance with the TAI technique.

Despite the similarity in CL size, it was also shown that low-AFC cows tended to have a higher P4 concentration 7 days after ovulation confirmation. It would be expected that a larger dominant follicle would result in a greater CL^33,34^. However, in *Bos indicus* cattle, there is evidence that CL size and luteotrophic capacity does not follow exactly the same pattern described in *Bos taurus*. For example, although CL size is a relevant factor, comparisons of luteal function between *Bos taurus* and *Bos indicus* cattle reveal that in *indicus* animals the ability of lutein cells to secrete P4 can be found even in smaller CL^35,36^. In addition, the proportion of large and small luteal cells that form the CL and the availability of high density lipoprotein in the animal may affect the steroidogenic CL capacity^37^. In the present study it was found that CL size was not associated with AFC, although a tendency to have higher P4 concentration was found in low AFC cows.

These positive aspects discussed certainly contributed to the greater pregnancy rate in the females with a very low count, which was approximately 12% greater than in the high AFC group. In *Bos indicus* (Nelore), Morotti et al.^8^ detected similar results, and Moraes et al.^7^ not only detected a better effect of a low AFC but also observed an interaction of AFC with the BCS: cows with a high AFC and a high BCS had the worst reproductive performance during the breeding season. In dairy cattle, Jimenez-Krassel et al.^4^ also showed productive and reproductive advantages for females with a low AFC, and a high count in females was associated with a shorter productive life within the herd and suboptimal fertility. All of these findings reinforce a controversial and highly complex issue regarding the relationship between AFC and fertility^12,13^, because comparing the pregnancy rate with rates reported in several studies conducted in *Bos taurus* beef and dairy animals reveals greater fertility for females with an AFC greater than 25 follicles^2,3,9,10,17,19,27,38^. On the other hand, at least from the Nelore *Bos indicus* cattle studies, it appears that a low AFC (< 15 follicles) leads to greater pregnancy rates when the cows are subjected to TAI programmes.

Despite the positive aspects for females with a low AFC, there was no difference in the ovulatory rate or ovulation time between groups. Similarly, other researchers have not observed differences in these reproductive parameters in Nelore cattle^8^. This fact reinforces the possibility that the differences related to TAI fertility are related not only to factors associated with follicular dynamics (dominant follicle diameter) but also to factors related to follicular cell development and oocyte competence, as revealed in the present study.

The transcriptional patterns of genes important for follicular cell development in the present study confirm this hypothesis by revealing that the low AFC group exhibited the upregulation of intercellular communication genes (oocytes: *BMP15*, *HAS2* and *EGFR*; cumulus cells: *EGFR*) and intercellular communication genes involved in meiotic control (*NPR2* and *NPR3* for both oocytes and cumulus cells). Intercellular communication, mediated by oocyte-secreted factors and EGF ligands, allows an oocyte to establish control of the proliferation and differentiation^39–41^, apoptosis^42^, luteinization^43^, metabolism^44^ and expansion^45^ of cumulus cells.

In the present study, females with a low AFC also showed the upregulation of genes related to epigenetic modulation and meiotic control (oocyte: *H1FOO* and cumulus cells: *RGS2*, *DNMT3A*, *IIDAC2* and *PAF1*), follicular growth (oocyte: *IGFBP2* and cumulus cells: *GSK3A*, *IGF1R* and *IGFBP2*), cell maintenance (oocyte: *GAPDH* and *NFKB2* and cumulus cells: *ATPL5*, *TFAM* and *PFKP*) and cellular stress response (oocyte: *HSF1* and cumulus cells: *HSP90AA1*, *HSPA5*, *HSPD1*, *SOD1* and *PRDX3*). On the other hand, although oocytes from the same AFC category showed lower expression of a gene related to cell stress and apoptosis (*XBP1*) than oocytes from the H-AFC group, cumulus cells from the low AFC group exhibited the upregulation of *CASP9*, *STAT3* and *XBP1*, genes that are also related to cell stress and apoptosis. Additionally, *TFAM*, a mitochondrial DNA binding protein (mtDNA) essential for the initiation of transcription and genome maintenance^46^, was highly expressed in the low AFC group. It has been shown that the primary role of TFAM is to maintain mtDNA integrity and that it is a key regulator of mtDNA copy number^47^ and contributes to the stress-mediated inflammatory response of mtDNA^48^. These differences suggest that oocyte and cumulus cells from females with a low or high AFC are undergoing differential epigenetic, cell growth and cellular stress processes. Further investigation is needed to elucidate the specific details of such differences.

The association of transcriptional patterns and AFC has been investigated previously in *Bos taurus* cross-breed females^11^, and a low AFC was associated with upregulated *CTSB* expression in cumulus cells, as well as a tendency for upregulated *CTSS* expression in cumulus cells and downregulated *AMH* and *TBC1D1* expression in granulosa and theca cells. Such results and the data described here are not directly comparable, as, in contrast to indicine, there is a well-established association of a low AFC and poor reproductive performance in taurine females both in vivo and in vitro. Not surprisingly, some results do not converge. For example, we reported decreased *ARO* expression in cumulus cells from animals with a low AFC, which was not observed in taurine females; instead, in taurine females, increased *ARO* expression was found in the granulosa cells, a cell type not assessed in this study.

All the contexts discussed here are highly relevant because although reproductive biotechniques have been developed for livestock, their use has been associated with epigenetic alterations^49^ due to the various management practices and environmental influencing factors to which the animals are submitted. The intensification of livestock has certainly generated greater nutritional, sanitary or behavioural/animal welfare challenges that somehow contributed to greater environmental stress on gametes and embryos, compromising the fertility of the herd^50–53^. Therefore, the present study also stands out as highly relevant due to the association of basic science with practical field results, revealing that a low AFC is associated with the high expression of genes responsible for cell maintenance, resistance to cell stress and apoptosis.

Although the processes of follicular growth and maturation depend largely on gonadotropins [luteinizing hormone (LH) and follicle stimulating hormone (FSH)], insulin-like growth factors (IGF1 and IGF2)^54,55^ are also considered of great importance because they assist in the proliferation and differentiation of granulosa and theca cells^56,57^. In addition, they act synergistically with gonadotropins to increase the expression of the genes that encode the steroidogenic enzymes^55,58^. IGFBP2, along with other IGF-binding proteins (IGFBP-1 to -7), are present in the blood and extracellular fluid and exhibit greater activity in the early stages of follicular development^59^. Yuan et al.^55^ revealed that changes in *IGFBP2* gene expression are opposite to those in *IGF1* or *IGF2* expression, i.e., in the dominant follicle, there is a decrease in *IGFBP2* mRNA and an increase in *IGF1* and *IGF2* mRNAs as dominance is achieved.

Finally, this study highlights the possibility that the AFC may affect the fertility of Nelore cattle subjected to a TAI programme, since females with a low AFC exhibited greater follicular diameters, tended to have higher P4 concentrations and a greater pregnancy rate. In addition, oocytes and cumulus cells from cattle in this group showed higher expression levels of genes linked to intercellular communication, meiotic control, epigenetic modulation, cellular stress response and follicular growth. These data are consistent with the fact that many proteins and growth factors are produced and stored in oocytes during follicular growth^60,61^ and oocyte competence is achieved as the dominant follicle grows^62^. Therefore, a follicular environment that results in larger dominant follicle diameters and more competent oocytes, with a positive effect on the subsequent luteal phase, may impact embryonic survival^63,64^ and thus benefit the fertility of beef cows in TAI.

## Conclusion

In conclusion, very low AFCs in Nelore cows resulted in a large dominant follicle diameter, a tendency to have higher progesterone concentration and greater pregnancy rate in TAI programmes. In addition, Nelore heifers with low AFCs exhibited oocytes and cumulus cells with distinct expression patterns of genes linked to intercellular communication, meiotic control, epigenetic modulation, adaptation and cellular stress response and follicular growth.

## Supplementary information

Supplementary Legends.

Supplementary Table 1.

Supplementary Table 2.

## Data Availability

We declare that all data generated in this study are available in the manuscript itself or in the supplementary files.

## References

[1] Burns DS, Jimenez-Krassel F, Ireland JL, Knight PG, Ireland JJ (2005). Numbers of antral follicles during follicular waves in cattle: Evidence for high variation among animals, very high repeatability in individuals, and an inverse association with serum follicle-stimulating hormone concentrations. Biol. Reprod..

[2] Evans AC (2012). Effects of maternal environment during gestation on ovarian folliculogenesis and consequences for fertility in bovine offspring. Reprod. Domest. Anim..

[3] Ireland JJ (2011). Does size matter in females? An overview of the impact of the high variation in the ovarian reserve on ovarian function and fertility, utility of anti-Mullerian hormone as a diagnostic marker for fertility and causes of variation in the ovarian reserve in cattle. Reprod. Fertil. Dev..

[4] Jimenez-Krassel F, Scheetz DM, Neuder LM, Pursley JR, Ireland JJ (2017). A single ultrasound determination of ≥ 25 follicles ≥ 3 mm in diameter in dairy heifers is predictive of a reduced productive herd life. J. Dairy Sci..

[5] Santos GMG (2016). High numbers of antral follicles are positively associated with in vitro embryo production but not the conception rate for FTAI in Nelore cattle. Anim. Reprod. Sci..

[6] Silva-Santos KC (2014). Antral follicle populations and embryo production in vitro and in vivo of *Bos indicus-taurus* donors from weaning to yearling ages. Reprod. Domest. Anim..

[7] Moraes FLZ, Morotti F, Costa CB, Lunardelli PA, Seneda MM (2019). Relationships between antral follicle count, body condition, and pregnancy rates after timed-AI in *Bos indicus* cattle. Theriogenology.

[8] Morotti F (2018). Ovarian follicular dynamics and conception rate in *Bos indicus* cows with different antral follicle counts subjected to timed artificial insemination. Anim. Reprod. Sci..

[9] Ireland JJ (2007). Follicle numbers are highly repeatable within individual animals but are inversely correlated with FSH concentrations and the proportion of good-quality embryos after ovarian stimulation in cattle. Hum. Reprod..

[10] Ireland JL (2008). Antral follicle count reliably predicts number of morphologically healthy oocytes and follicles in ovaries of young adult cattle. Biol. Reprod..

[11] Ireland J (2009). Variation in the ovarian reserve is linked to alterations in intrafollicular estradiol production and ovarian biomarkers of follicular differentiation and oocyte quality in cattle. Biol. Reprod..

[12] Morotti F (2015). Is the number of antral follicles an interesting selection criterium for fertility in cattle. Anim. Reprod..

[13] Morotti F (2017). Antral follicle count in cattle: Advantages, challenges, and controversy. Anim. Reprod..

[14] Zangirolamo AF, Morotti F, da Silva NC, Sanches TK, Seneda MM (2018). Ovarian antral follicle populations and embryo production in cattle. Anim. Reprod..

[15] Seneda MM (2019). Antral follicle population in prepubertal and pubertal heifers. Reprod. Fertil. Dev..

[16] Morotti F (2017). Correlation between phenotype, genotype and antral follicle population in beef heifers. Theriogenology.

[17] Mossa F (2012). Low numbers of ovarian follicles ≥ 3mm in diameter are associated with low fertility in dairy cows. J. Dairy Sci..

[18] Singh J, Dominguez M, Jaiswal R, Adams GP (2004). A simple ultrasound test to predict the superstimulatory response in cattle. Theriogenology.

[19] Jimenez-Krassel F (2009). Evidence that high variation in ovarian reserves of healthy young adults has a negative impact on the corpus luteum and endometrium during estrous cycles in cattle. Biol. Reprod..

[20] Pontes JH (2009). Comparison of embryo yield and pregnancy rate between in vivo and in vitro methods in the same Nelore (*Bos indicus*) donor cows. Theriogenology.

[21] Sa Filho MF, Crespilho AM, Santos JE, Perry GA, Baruselli PS (2010). Ovarian follicle diameter at timed insemination and estrous response influence likelihood of ovulation and pregnancy after estrous synchronization with progesterone or progestin-based protocols in suckled *Bos indicus* cows. Anim. Reprod. Sci..

[22] Pfeifer LFM, Leal SDCBDS, Schneider A, Schmitt E, Corrêa MN (2012). Effect of the ovulatory follicle diameter and progesterone concentration on the pregnancy rate of fixed-time inseminated lactating beef cows. R. Bras. Zootec..

[23] Pfeifer LF (2015). Timed artificial insemination in blocks: A new alternative to improve fertility in lactating beef cows. Anim. Reprod. Sci..

[24] Ginther OJ, Knopf L, Kastelic JP (1989). Temporal associations among ovarian events in cattle during oestrous cycles with two and three follicular waves. J. Reprod. Fertil..

[25] Figueiredo RA, Barros CM, Pinheiro OL, Soler JM (1997). Ovarian follicular dynamics in Nelore breed (*Bos indicus*) cattle. Theriogenology.

[26] Fontes PK, Castilho ACS, Razza EM, Nogueira MFG (2020). Bona fide gene expression analysis of samples from the bovine reproductive system by microfluidic platform. Anal. Biochem..

[27] Martinez MF, Sanderson N, Quirke LD, Lawrence SB, Juengel JL (2016). Association between antral follicle count and reproductive measures in New Zealand lactating dairy cows maintained in a pasture-based production system. Theriogenology.

[28] Meneghetti M, Sa Filho OG, Peres RF, Lamb GC, Vasconcelos JL (2009). Fixed-time artificial insemination with estradiol and progesterone for *Bos indicus* cows I: Basis for development of protocols. Theriogenology.

[29] Perry GA (2005). Relationship between follicle size at insemination and pregnancy success. Proc. Natl. Acad. Sci. U. S. A..

[30] Nogueira E (2019). Timed artificial insemination plus heat I: Effect of estrus expression scores on pregnancy of cows subjected to progesterone-estradiol-based protocols. Animal.

[31] Gimenes LU (2008). Follicle deviation and ovulatory capacity in *Bos indicus* heifers. Theriogenology.

[32] Sa Filho OG, Meneghetti M, Peres RF, Lamb GC, Vasconcelos JL (2009). Fixed-time artificial insemination with estradiol and progesterone for *Bos indicus* cows II: Strategies and factors affecting fertility. Theriogenology.

[33] Vasconcelos JL, Sartori R, Oliveira HN, Guenther JG, Wiltbank MC (2001). Reduction in size of the ovulatory follicle reduces subsequent luteal size and pregnancy rate. Theriogenology.

[34] Baruselli PS (2012). Manipulation of follicle development to ensure optimal oocyte quality and conception rates in cattle. Reprod. Domest. Anim..

[35] Carvalho JB (2008). Effect of early luteolysis in progesterone-based timed AI protocols in *Bos indicus*, *Bos indicus*×*Bos taurus*, and *Bos taurus heifers*. Theriogenology.

[36] Sartori R, Barros CM (2011). Reproductive cycles in *Bos indicus* cattle. Anim. Reprod. Sci..

[37] Wiltbank MC (2012). Comparison of endocrine and cellular mechanisms regulating the corpus luteum of primates and ruminants. Anim. Reprod..

[38] Jimenez-Krassel F (2015). Concentration of anti-Mullerian hormone in dairy heifers is positively associated with productive herd life. J. Dairy Sci..

[39] Li R, Norman RJ, Armstrong DT, Gilchrist RB (2000). Oocyte-secreted factor(s) determine functional differences between bovine mural granulosa cells and cumulus cells. Biol. Reprod..

[40] Gilchrist RB, Ritter LJ, Armstrong DT (2004). Oocyte-somatic cell interactions during follicle development in mammals. Anim. Reprod. Sci..

[41] Calder MD, Caveney AN, Sirard MA, Watson AJ (2005). Effect of serum and cumulus cell expansion on marker gene transcripts in bovine cumulus-oocyte complexes during maturation in vitro. Fertil. Steril..

[42] Hussein TS, Froiland DA, Amato F, Thompson JG, Gilchrist RB (2005). Oocytes prevent cumulus cell apoptosis by maintaining a morphogenic paracrine gradient of bone morphogenetic proteins. J. Cell Sci..

[43] Eppig JJ (1997). Oocyte control of granulosa cell development: How and why. Hum. Reprod..

[44] Eppig JJ, Pendola FL, Wigglesworth K, Pendola JK (2005). Mouse oocytes regulate metabolic cooperativity between granulosa cells and oocytes: Amino acid transport. Biol. Reprod..

[45] Vanderhyden BC, Caron PJ, Buccione R, Eppig JJ (1990). Developmental pattern of the secretion of cumulus expansion-enabling factor by mouse oocytes and the role of oocytes in promoting granulosa cell differentiation. Dev. Biol..

[46] de Oliveira VC (2019). Edition of TFAM gene by CRISPR/Cas9 technology in bovine model. PLoS ONE.

[47] Hallberg BM, Larsson NG (2011). TFAM forces mtDNA to make a U-turn. Nat. Struct. Mol. Biol..

[48] Kang I, Chu CT, Kaufman BA (2018). The mitochondrial transcription factor TFAM in neurodegeneration: Emerging evidence and mechanisms. FEBS Lett..

[49] Urrego R, Rodriguez-Osorio N, Niemann H (2014). Epigenetic disorders and altered gene expression after use of Assisted Reproductive Technologies in domestic cattle. Epigenetics.

[50] Lucy MC (2019). Stress, strain, and pregnancy outcome in postpartum cows. Anim. Reprod..

[51] Collier RJ, Renquist BJ, Xiao Y (2017). A 100-year review: Stress physiology including heat stress. J. Dairy Sci..

[52] Michael J, Baruselli PS, Campanile G (2019). Influence of nutrition, body condition, and metabolic status on reproduction in female beef cattle: A review. Theriogenology.

[53] Alfieri AA, Leme RA, Agnol AMD, Alfieri AF (2019). Sanitary program to reduce embryonic mortality associated with infectious diseases in cattle. Anim. Reprod..

[54] Monniaux D, Monget P, Besnard N, Huet C, Pisselet C (1997). Growth factors and antral follicular development in domestic ruminants. Theriogenology.

[55] Yuan W, Bao B, Garverick HA, Youngquist RS, Lucy MC (1998). Follicular dominance in cattle is associated with divergent patterns of ovarian gene expression for insulin-like growth factor (IGF)-I, IGF-II, and IGF binding protein-2 in dominant and subordinate follicles. Domest. Anim. Endocrinol..

[56] Spicer LJ, Echternkamp SE (1995). The ovarian insulin and insulin-like growth factor system with an emphasis on domestic animals. Domest. Anim. Endocrinol..

[57] Giudice LC (1992). Insulin-like growth factors and ovarian follicular development. Endocr. Rev..

[58] deMoura MD, Choi D, Adashi EY, Payne DW (1997). Insulin-like growth factor-I-mediated amplification of follicle-stimulating hormone-supported progesterone accumulation by cultured rat granulosa cells: enhancement of steroidogenic enzyme activity and expression1. Biol. Reprod..

[59] Mazerbourg S, Monget P (2018). Insulin-like growth factor binding proteins and IGFBP proteases: A dynamic system regulating the ovarian folliculogenesis. Front. Endocrinol..

[60] Gandolfi TA, Gandolfi F (2001). The maternal legacy to the embryo: Cytoplasmic components and their effects on early development. Theriogenology.

[61] Reader KL, Stanton JL, Juengel JL (2017). The role of oocyte organelles in determining developmental competence. Biology (Basel)..

[62] Arlotto T, Schwartz JL, First NL, Leibfried-Rutledge ML (1996). Aspects of follicle and oocyte stage that affect in vitro maturation and development of bovine oocytes. Theriogenology.

[63] Santos JE, Thatcher WW, Chebel RC, Cerri RL, Galvao KN (2004). The effect of embryonic death rates in cattle on the efficacy of estrus synchronization programs. Anim. Reprod. Sci..

[64] Mann GE, Lamming GE (2001). Relationship between maternal endocrine environment, early embryo development and inhibition of the luteolytic mechanism in cows. Reproduction.

