# Supplementary material for: Ovarian follicular dynamics, progesterone concentrations, pregnancy rates and transcriptional patterns in Bos indicus females with a high or low antral follicle count
Source: Sci Rep. 2020 Nov 11;10:19557. doi: 10.1038/s41598-020-76601-5 (PMC7658257; doi:10.1038/s41598-020-76601-5)
Supplement: Supplementary file 1 — Supplementary Legends. [file 41598_2020_76601_MOESM1_ESM.docx]

**Ovarian follicular dynamics, progesterone concentrations, pregnancy rates and transcriptional patterns in *Bos indicus* females with a high or low antral follicle count**

Marina Amaro de Lima^1,#^, Fábio Morotti^2,#*^, Bernardo Marcozzi Bayeux^3^, Rômulo Germano de Rezende^3^, Ramon Cesar Botigelli^4^, Tiago Henrique Camara De Bem^1^, Patrícia Kubo Fontes^4^, Marcelo Fábio Gouveia Nogueira^5^, Flávio Vieira Meirelles^1^, Pietro Sampaio Baruselli^3^, Juliano Coelho da Silveira^1^, Felipe Perecin^1,§^, Marcelo Marcondes Seneda^2,§^

**Supplementary material**

**Supplementary Table 1 -** TaqMan Assays used to assess gene expression in the microfluidic platform.

**Supplementary Table 2 -** Relative expression levels in oocytes and cumulus cells in Nelore heifers with a low antral follicle count (L-AFC) or high AFC (H-AFC). Expression levels were calculated using *PPIA* as the reference gene. Although gene expression was assessed in 10 samples (animals) for each group, the column titled “N” refers to the number of samples in which expression was detected. When none of the samples showed expression of a given gene “n.d.” (not detected) is indicated. A dash (-) for the standard deviation (S.D.) or P-value indicates that the value was not calculated.
